# Tanshinone IIA Combined With Cyclosporine A Alleviates Lung Apoptosis Induced by Renal Ischemia-Reperfusion in Obese Rats

**DOI:** 10.3389/fmed.2021.617393

**Published:** 2021-05-03

**Authors:** He Tai, Xiao-lin Jiang, Nan Song, Hong-he Xiao, Yue Li, Mei-jia Cheng, Xiao-mei Yin, Yi-ran Chen, Guan-lin Yang, Xiao-yu Jiang, Jin-song Kuang, Zhi-ming Lan, Lian-qun Jia

**Affiliations:** ^1^Key Laboratory of Ministry of Education for Traditional Chinese Medicine Viscera-State Theory and Applications, Liaoning University of Traditional Chinese Medicine, Shenyang, China; ^2^Department of Nephrology, The Fourth of Affiliated Hospital of Guangzhou University of Traditional Chinese Medicine (Shenzhen Traditional Chinese Medicine Hospital), Guangzhou University of Traditional Chinese Medicine, Shenzhen, China; ^3^School of Pharmacy, Liaoning University of Traditional Chinese Medicine, Dalian, China; ^4^Department of Foreign Languages, Dalian Medical University, Dalian, China; ^5^Department of Endocrinology and Metabolism, The Fourth People's Hospital of Shenyang, Shenyang, China; ^6^Department of Medical Laboratory, The Fourth Affiliated Hospital of Guangzhou University of Traditional Chinese Medicine (Shenzhen Traditional Chinese Medicine Hospital), Guangzhou University of Traditional Chinese Medicine, Shenzhen, China

**Keywords:** renal ischemia-reperfusion, obesity, mitochondrial dysfunction, acute lung injury, tanshinone IIA, cyclosporine A

## Abstract

Acute lung injury (ALI), which is induced by renal ischemia-reperfusion (IR), is one of the leading causes of acute renal IR-related death. Obesity raises the frequency and severity of acute kidney injury (AKI) and ALI. Tanshinone IIA (TIIA) combined with cyclosporine A (CsA) was employed to lessen the lung apoptosis led by renal IR and to evaluate whether TIIA combined with CsA could alleviate lung apoptosis by regulating mitochondrial function through the PI3K/Akt/Bad pathway in obese rats. Hematoxylin-eosin (HE) staining was used to assess the histology of the lung injury. Terminal deoxynucleotidyl transferase-mediated dUTP nick end-labeling (TUNEL) was used to assess apoptosis of the lung. Electron microscopy was used to assess mitochondrial morphology in lung cells. Arterial blood gas and pulmonary function were used to assess the external respiratory function. Mitochondrial function was used to assess the internal respiratory function and mitochondrial dynamics and biogenesis. Western blot (WB) was used to examine the PI3K/Akt/Bad pathway-related proteins. TIIA combined with CsA can alleviate lung apoptosis by regulating mitochondrial function through the PI3K/Akt/Bad pathway in obese rats.

## Introduction

Acute kidney injury (AKI), as a common complication, is very serious, and even life-threatening (high mortality) in critically patients (1). The major cause of AKI is renal ischemia-reperfusion (IR) (2). Obesity is closely correlated with metabolic syndromes, including hyperuricemia, hyperlipidemia, and diabetes, and they can lead to hypertension and pathoglycemia and can all be seen as chronic hyperinflammatory conditions (3), which increase the severity and morbidity related to kidney disease (4). Ischemia AKI is often combined with multiple-organ dysfunction. It is worth noting that respiratory failure rather than kidney failure is the main cause of death induced by AKI (5). The pathogenesis of acute lung injury (ALI) led by acute renal IR is ambiguous, which could be related to renal dysfunction-induced overload volume and hyperinflammatory state-induced lung injury (6). Lung endothelial cell inflammation and apoptosis induce ALI following AKI induced by renal IR (7, 8). As a potential mediator between lung and kidney injury, pulmonary microvascular endothelial cells (PMVECs) express proapoptosis and proinflammation genes, which can change after AKI is induced by renal IR (7, 8). Numerous studies have demonstrated that the occurrence of IR (especially in the nerves and heart) is closely related with mitochondrial dysfunction (9, 10). However, to our knowledge, no study has explored the change of mitochondrial function in the lung following renal IR in obese rats.

As the main active ingredient of the *Salvia miltiorrhiza* Bge, the main biological activities of tanshinone IIA (TIIA) are reducing the inflammatory response and resisting oxidant stress (11). Additionally, TIIA has a protective effect against myocardial ischemia (12). TIIA relieved acute lung injury induced by lipopolysaccharide *via* reducing proinflammatory factors and TRPM7 (13). In the meantime, TIIA performed a protective role in AKI induced by folic acid (14). Several studies have shown that TIIA can inhibit mitochondrial permeability transition pore (mPTP) and then achieve the aims of cardioprotection (15) and liver protection (16). With its anticalcineurin properties, cyclosporine A (CsA) can bind to cyclophilin D (CyP-D), preventing mPTP opening, and thereby decreasing the injury due to IR (17). CsA injection before ischemia can be used to preserve renal function (18). There are few studies available on TIIA and CsA lung protection through preventing mPTP opening.

Phosphoinositide-3 kinase (PI3K) transduces survival effects, which depend on the Akt kinase phosphorylation and activation, followed by proapoptotic Blc-2 family protein (Bad) phosphorylation and inhibition. PI3K plays a significant role in growth factor signal transduction. Under various cytokines and the activation of physiochemical factors, PI3K can produce myoinositol as a second messenger, and Akt performs crucial roles in many biological processes, including cell metabolism, cell cycle, cell growth, and apoptosis (19). The PI3K/Akt/Bad signaling pathway performs a significant function in inhibiting mitochondria-mediated apoptosis (20). However, there have been no studies on TIIA and CsA pulmonary protection with correcting mitochondrial dysfunction through the PI3K/Akt/Bad signal pathway in obese rats.

Given the increasing epidemic of obesity, especially in old people, the current study aimed to evaluate a method for AKI (induced by IR)-induced lung mitochondrial dysfunction using a combination of TIIA and CsA to cross the gap between mitochondrial dysfunction and lung injury, for the purpose of finding new therapeutic targets.

## Results

### Causes of Death During the Experiment

Ultimately, 66 rats [Sham group (13 rats), IR group (13 rats), IR (obese) group (10 rats), TIIA group (10 rats), CsA group (11 rats), and TIIA+CsA group (9 rats)] finished the study, and 54 rats died during the study (Table 1).

**Table 1:** The cause of death in the six groups rats.

**Cause of death**	**Sham**	**IR (non)**	**IR (obese)**	**TIIA**	**CsA**	**TIIA+CsA**
Infection after injection	00	0	0	1	0	0
Massive hemorrhage	1	1	2	2	3	3
Infection after surgery	2	2	3	3	2	3
Intestinal obstruction	4	4	5	4	4	5
Number of completed cases [*n* (%)]	13 (65.00%)	13 (65.00%)	10 (50.00%)	10 (50.00%)	11 (55.00%)	9 (45.00%)

### TIIA Combined With CsA Improved the Arterial Blood Gas and Pulmonary Function Led by Acute Renal IR in Obese Rats

**Figure 1:**
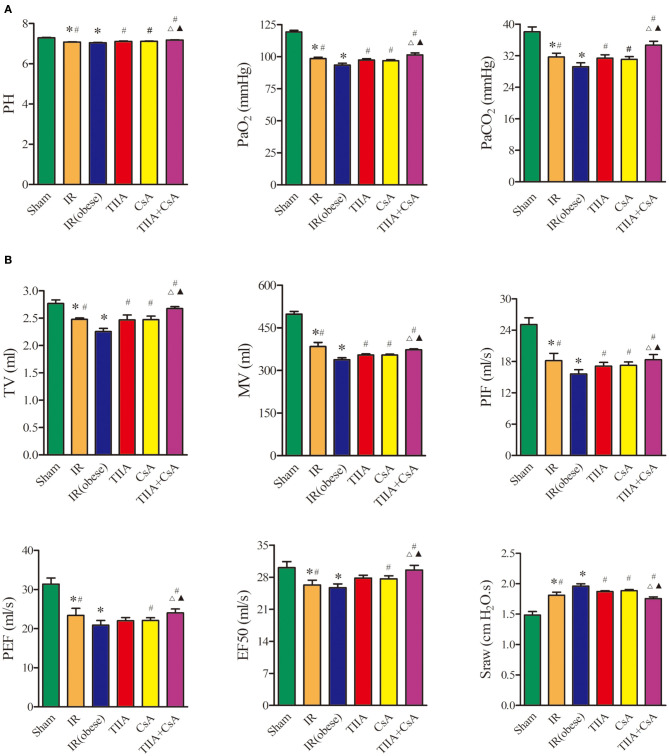
Tanshinone IIA (TIIA)+ cyclosporine A (CsA) improved the arterial blood gas and pulmonary function induced by renal ischemia-reperfusion (IR). Rats were pretreated with TIIA alone or in combination with CsA followed by removing the right kidney and clamping of the left renal artery for 30 min and reperfusion for 24 h. Sham rats were used as control. Representative arterial blood gas **(A)** and pulmonary function **(B)** were evaluated under different conditions. The scale bars represent a length of 200 μm on histology. Data are shown as mean ± SD. *p < 0.05 vs. sham group, ^#^*p* < 0.05 vs. IR (obese) group, ^Δ^*p* < 0.05 vs. TIIA group, ^▴^*p* < 0.05 vs. CsA group.

We used arterial blood gas (Figure 1A) and pulmonary function (Figure 1B), which are external indicators of respiratory function. IR and IR (obese) can both decrease the blood pH, arterial partial pressure of carbon dioxide (PaCO_2_), and arterial partial pressure of oxygen (PaO_2_) especially IR (obese) (*p* < 0.05), and they can be increased by TIIA, CsA, and TIIA+CsA (*p* < 0.05), while pretreatment with TIIA+CsA was higher than TIIA and CsA (*p* < 0.05; Figure 1A). Rat pulmonary function was tested to further assess the lung injury. The TV, MV, PIF, PEF, and EF50 were decreased by IR and IR (obese), especially the IR (obese) (*p* < 0.05), which were decreased by TIIA, CsA, and TIIA+CsA (*p* < 0.05), and pretreatment with TIIA+CsA was higher than TIIA and CsA (*p* < 0.05). The Sraw was increased by IR and IR (obese), especially in the IR (obese) (*p* < 0.05), which were upregulated by TIIA, CsA, and TIIA+CsA (*p* < 0.05), and pretreatment with TIIA+CsA was lower than TIIA and CsA (*p* < 0.05; Figure 1B).

### TIIA Combined With CsA Improved the Lungs Pathological Structure Led by Acute Renal IR in Obese Rats

**Figure 2:**
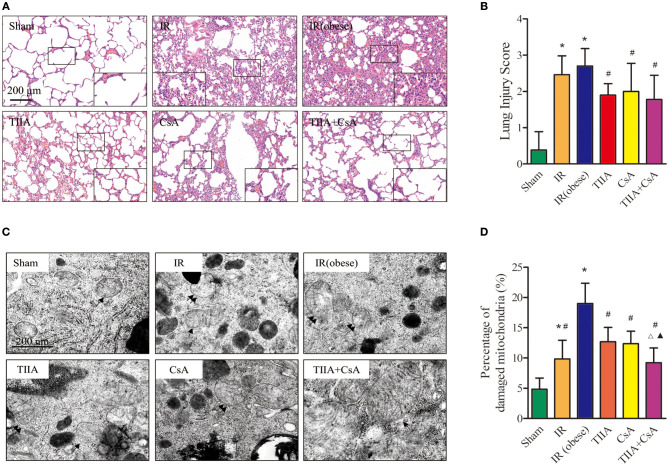
Tanshinone IIA (TIIA)+ cyclosporine A (CsA) preserved lung architecture in renal ischemia-reperfusion (IR)-induced lung injury. Rats were pretreated with TIIA alone or in combination with CsA followed by removing the right kidney and clamping of the left renal artery for 30 min and reperfusion for 24 h. Sham rats were used as control. Representative photomicrographs of lung histology **(A)**, the scale bars represent a length of 200 μm on histology, and lung injury scores **(B)** were evaluated under different conditions. The scale bars represent a length of 200 μm on histology. Electron microscope pictures (×40,000) of rat lung tissue after renal ischemia-reperfusion (IR). Abnormal mitochondrial (paired black arrow) morphology showed mitochondrial membrane rupture or swellings; normal mitochondrial (single black arrow) morphology type showed smooth mitochondrial membrane and smooth and distinct inner carinulae **(C)**, and percentage of damaged mitochondria **(D)**. Data are shown as mean ± SD. *p < 0.05 vs. sham group, ^#^*p* < 0.05 vs. IR (obese) group, ^Δ^*p* < 0.05 vs. TIIA group, ^▴^*p* < 0.05 vs. CsA group.

We observed lung histology employing hematoxylin-eosin (HE) staining (Figure 2A). The alveoli only had slight exudation in the Sham group. Obviously, IR and IR (obese) can increase the numbers of disordered alveoli, especially IR (obese), with large numbers of inflammatory cells and blood cells in the alveolar cavity, combined with an obvious pulmonary interstitial edema. However, using TIIA, CsA, and TIIA+CsA could alleviate lung injury, shown as an improvement in interstitial edema and a reduction in inflammatory cells and red cells in the alveoli. All the changes associated with injury were evaluated through histological scores (Figure 2B). Statistical results showed that the IR and IR (obese) could increase the lung injury scores compared with the Sham (*p* < 0.05), and TIIA, CsA, or TIIA+CsA could decrease the injury scores (*p* < 0.05; Figure 2B).

### Lung Mitochondrial Morphological Changes Led by Acute Renal IR in Obese Rats

We detect the mitochondrial function, which can be thought as an internal indicator of respiratory function. First, we used electron microscopy to observe the lung mitochondrial morphological changes (Figure 2).

Electron microscopy images (×40,000) of rat lung tissue showed that the alveolar type II epithelial cells of the IR and IR (obese) groups showed the abnormal mitochondrial morphology in the form of swelling, even membrane rupture (paired black arrow) following renal IR, and most of mitochondria showed the normal morphology indicators (single black arrow) in the sham group. Renal IR and IR (obese) could increase the percentage of damaged mitochondria compared with the Sham group (*p* < 0.05), especially in the IR (obese), and giving TIIA, CsA, and TIIA+CsA could decrease the percentage of damaged mitochondria (*p* < 0.05), and pretreatment with TIIA+CsA was lower than TIIA and CsA (*p* < 0.05; Figures 2C,D).

### TIIA Combined With CsA Reduced Lung Apoptosis Led by Acute Renal IR in Obese Rats

**Figure 3:**
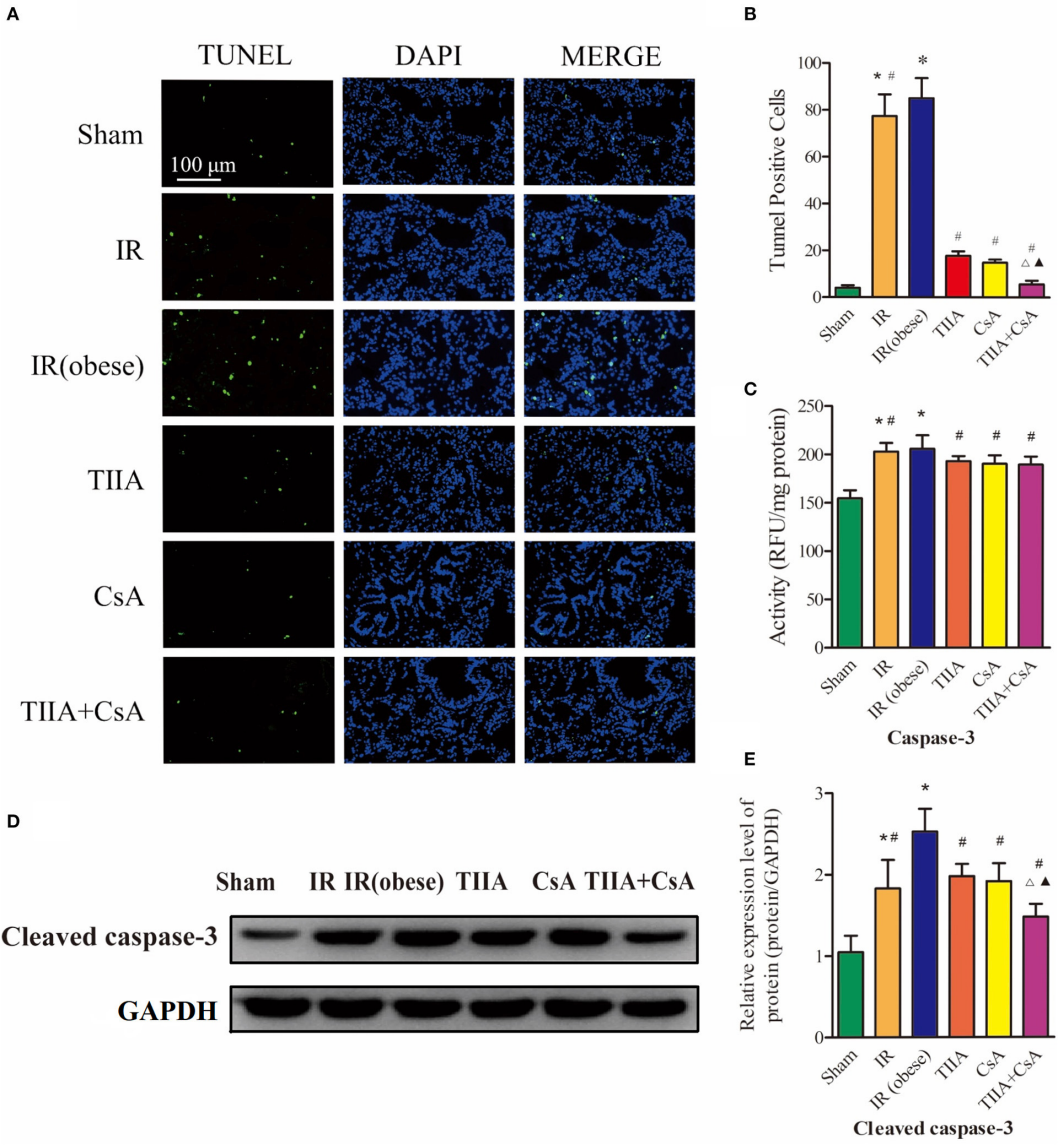
Tanshinone IIA (TIIA)+ cyclosporine A (CsA) inhibited lung cells apoptosis after renal ischemia-reperfusion (IR). **(A)** Lung apoptosis with TUNEL; **(B)** TUNEL-positive cells in lung; **(C)** caspase-3 activity. Rats were pretreated with TIIA alone or in combination with CsA followed by removing the right kidney and clamping of the left renal artery for 30 min and reperfusion for 24 h. Sham rats were used as control. Representative apoptosis of lung cells **(A)** and TUNEL-positive cells **(B)** were evaluated under different conditions. The scale bars represent a length of 100 μm on histology. The activity of myocardial caspase-3 **(C)**, the protein expression of cleaved caspase-3 **(D)**, and the protein expression of cleaved caspase-3 **(E)** were evaluated in different groups. Data are shown as mean ± SD. *p < 0.05 vs. sham group, ^#^*p* < 0.05 vs. IR (obese) group, ^Δ^*p* < 0.05 vs. TIIA group, ^▴^*p* < 0.05 vs. CsA group.

To research the effect of TIIA combined with CsA on lung cell apoptosis led by acute renal IR in obese rats, TUNEL assay was used to assess lung cell apoptosis (Figure 3A). The apoptotic cells of lung tissue in the two groups [IR (obese) and IR] were obviously increased, especially IR (obese) group (*p* < 0.05). However, giving CsA, TIIA, CsA, and TIIA+CsA alleviated lung cell apoptosis (*p* < 0.05), and pretreatment with TIIA+CsA was lower than TIIA and CsA (*p* < 0.05; Figure 3B). IR (obese) and IR could activate the caspase-3, especially IR (obese) (*p* < 0.05), while giving TIIA, CsA, and TIIA+CsA could decrease caspase-3 activity in lung cells (*p* < 0.05; Figure 3C). The method of western blot was used to detect the cleaved caspase-3 (Figure 3D), which was increased by IR (obese) and IR, especially IR (obese) (*p* < 0.05). However, giving TIIA and CsA could decrease the protein level of cleaved caspase-9/3, and pretreatment with TIIA+CsA was lower than TIIA and CsA (*p* < 0.05; *p* < 0.05; Figure 3E).

### TIIA Combined With CsA Can Improve Mitochondrial Dysfunction Led by Acute Renal IR in Obese Rats

**Figure 4:**
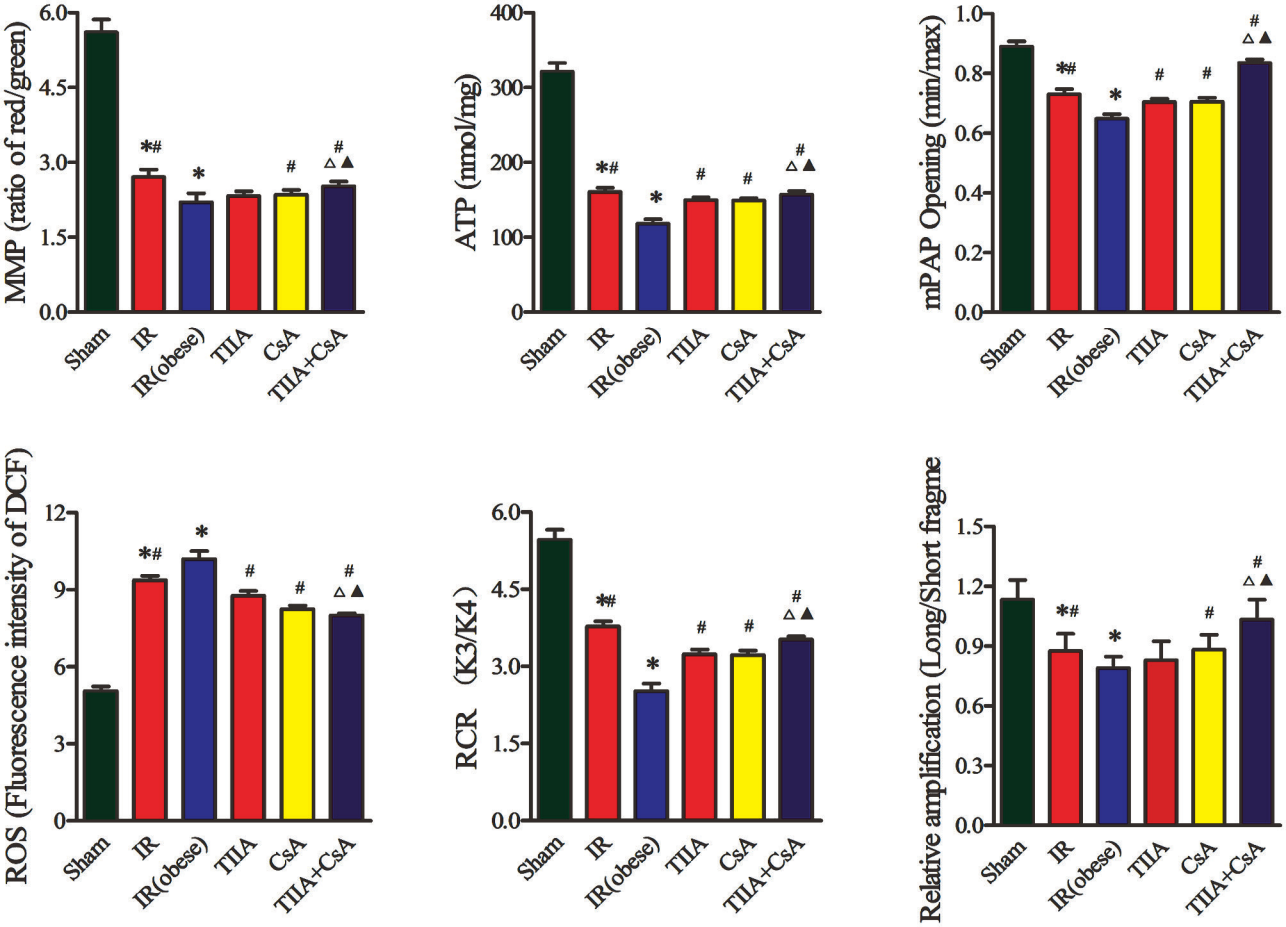
Tanshinone IIA (TIIA)+ cyclosporine A (CsA) preserved lung mitochondrial function in renal ischemia-reperfusion (IR)-induced lung injury. The MMP (ratio of red/green), the mitochondrial ATP, the opening of mPAP (%), the mitochondrial ROS, the mitochondrial RCR, and the mtDNA damage (ratio of long/short fragments) were recorded above. Rats were pretreated with TIIA alone or in combination with CsA followed by removing the right kidney and clamping of the left renal artery for 30 min and reperfusion for 24 h. Sham rats were used as control. *p < 0.05 vs. sham group, ^#^*p* < 0.05 vs. IR (obese) group, ^Δ^*p* < 0.05 vs. TIIA group, ^▴^*p* < 0.05 vs. CsA group.

We detected the RCR, mitochondrial ROS, ATP, MMP (ratio of red/green), the opening of mPAP (%), and the mtDNA to evaluate the mitochondrial function in lung tissue. Mitochondria of lung tissues were separated from rat lung tissues. Acute renal IR can increase the ROS level and the opening of mPAP (%) dramatically, especially in the IR (obese) (*p* < 0.05), ROS level and the opening of mPAP (%) can be decreased by giving CsA, TIIA, and TIIA+CsA (*p* < 0.05), and pretreatment with TIIA+CsA was lower than TIIA and CsA (*p* < 0.05). Mitochondrial RCR, ATP level, and the MMP (ratio of red/green) could be decreased by acute renal IR dramatically, especially the IR (obese) (*p* < 0.05), which could be increased by giving CsA, TIIA, and TIIA+CsA (*p* < 0.05), and pretreatment with TIIA+CsA was higher than TIIA and CsA (*p* < 0.05). We used real-time qPCR to evaluate the levels of mtDNA damage. IR (obese) and IR decreased ratios of long/short fragments, especially IR (obese) (*p* < 0.05), which could be increased by giving CsA, TIIA, and TIIA+CsA (*p* < 0.05), and pretreatment with TIIA+CsA was higher than TIIA and CsA (*p* < 0.05; Figure 4).

### TIIA Combined With CsA Can Improve the Abnormity of Mitochondrial Biogenesis and Dynamics Induced by Acute Renal IR in Obese Rats

**Figure 5:**
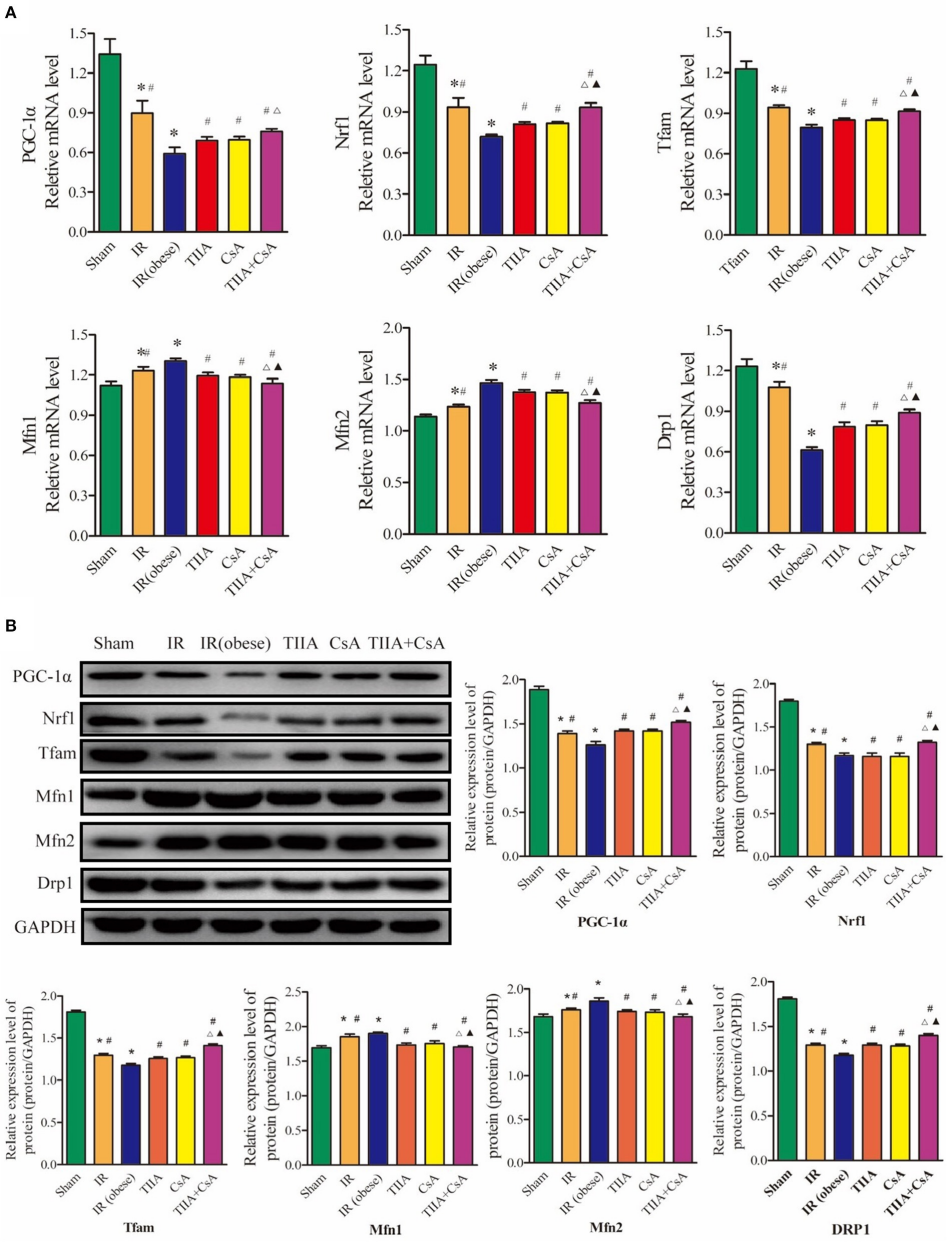
Tanshinone IIA (TIIA)+ cyclosporine A (CsA) preserved lung mitochondrial biogenesis and dynamics in renal renal ischemia-reperfusion (IR)-induced lung injury. The expression of PGC-1α, Nrf1, and Tfam in mRNA **(A)** and protein **(B)** levels. The expression of Mfn1, Mfn2, and Drp1 in mRNA level. *p < 0.05 vs. sham group, #*p* < 0.05 vs. IR (obese) group, ▵*p* < 0.05 vs. TIIA group, ▴*p* < 0.05 vs. CsA group.

Real-time qPCR and western blot were used to measure the mitochondrial dynamics and biogenesis, and we chose PGC-1α, Nrf1, and Tfam to represent mitochondrial biogenesis and Drp1 (fission), Mfn1 (fusion), and Mfn2 (fusion) to stand for mitochondrial dynamics (fission and fusion courses). In our study, the results showed that IR (obese) and IR could decrease the mRNA and protein levels of PGC-1α, Nrf1, Tfam, and Drp1, especially in the IR (obese) (*p* < 0.05), which were increased by giving with CsA, TIIA, and TIIA+CsA, and pretreatment with TIIA+CsA was higher than TIIA and CsA (*p* < 0.05; *p* < 0.05). IR (obese) and IR could increase the mRNA and protein expression levels of Mfn1 and Mfn2, especially IR (obese) (*p* < 0.05), which can be decreased by giving CsA, TIIA, and TIIA+CsA (*p* < 0.05), and pretreatment with TIIA+CsA was lower than TIIA and CsA (*p* < 0.05; Figures 5A,B).

### TIIA Combined With CsA Modulated the PI3K/Akt/Bad Pathway

**Figure 6:**
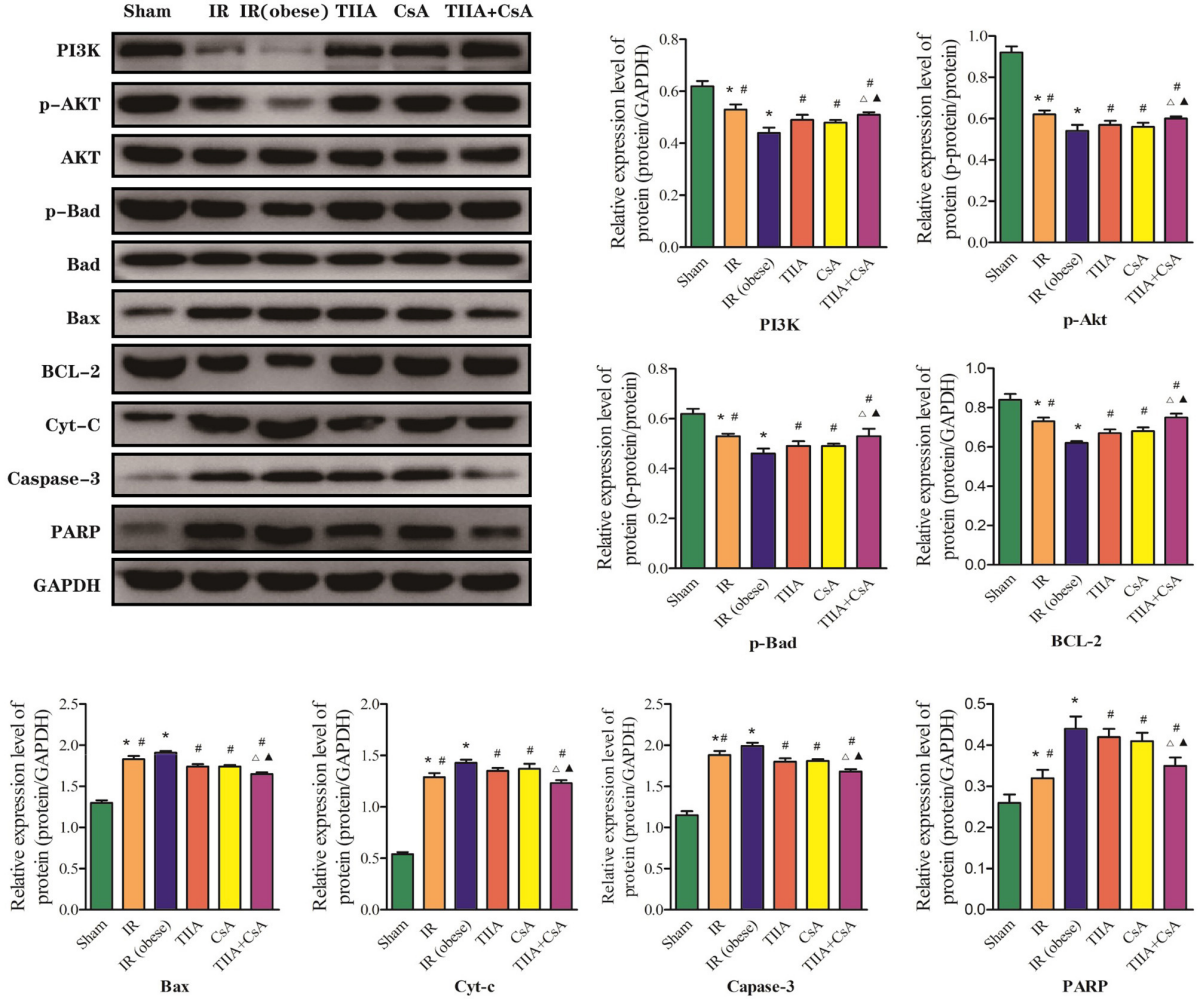
Tanshinone IIA (TIIA)+ cyclosporine A (CsA) modulated myocardial PI3K/Akt/Bad pathway. The expression of PI3K, p-Akt, Akt, p-Bad, Bad, Bax, Bcl-2, Cyt-c, caspase-3, and PARP in protein level. *p < 0.05 vs. sham group, ^#^*p* < 0.05 vs. IR (obese) group, ^Δ^*p* < 0.05 vs. TIIA group, ^▴^*p* < 0.05 vs. CsA group.

Finally, western blot was used to measure the target proteins of the PI3K/Akt/Bad pathway. We detected the PI3K, Akt, p-Akt, Bad, p-Bad, Bcl-2, Bax, Cyt-c, caspase-3, and PARP protein expression levels. The protein expression levels of Bax, Cyt-c, caspase-3, and PARP could be dramatically increased by acute renal IR, especially IR (obese) (*p* < 0.05), which could be decreased by giving CsA, TIIA, and TIIA+CsA (*p* < 0.05), and pretreatment with TIIA+CsA was lower than TIIA and CsA (*p* < 0.05). TIIA, CsA, and TIIA+CsA could induce Akt phosphorylation and enhance PI3K, p-Akt, p-Bad, and Bcl-2 expression and downregulate expression of p-Bad/Bad, Cyt-c, caspase-3, and PARP, especially TIIA+CsA (*p* < 0.05; Figure 6).

## Discussion

AKI often induces ALI in critically ill patients. When AKI is combined with ALI, the mortality is as high as ~80% (21). As a clear risk factor for cardiovascular disease, hyperlipidemia can increase the risk of renal I/R injury by increasing ROS and inflammation (22, 23). In our study, we used high-fat diet (HFD) to feed rats to establish an obesity model. HFD cannot induce a significant injury in renal function and histological structure but can increase nuclear translocation of transcription factors (e.g., NF-κB) and their activity; these effects in turn could worsen the inflammatory damage in an autocrine-dependent manner (23). Renal IR triggers many complex changes that eventually induce apoptosis and necrosis in renal tissues. Inflammation and ROS can be increased by oxidative stress, inducing proinflammatory mediator release during the phase of reperfusion. For the mechanism of renal IR described above, several anti-inflammatory and antioxidant agents were discovered to play effective roles in reducing the injury of renal IR. They can raise the survivability of the kidney induced by IR injury (24). AKI often goes with ALI in critically ill patients, and there is a clear relationship between AKI and ALI, but specific pathophysiological change between the kidney and the lungs remains unclear. This phenomenon has recently become a hot issue because of abnormal results; despite the use of dialysis, the mortality of AKI has remained at 50% (25), therefore, we must search for a new method to reduce the mortality of AKI combined with obesity. During 6 h of renal injury, pulmonary vascular permeability and associated lung diseases are increased due to oxidative stress and inflammation (5). Using multiple lines, White et al. found that apoptosis induced lung injury at 24 h (7). In our study, renal ischemia-reperfusion injury (IRI) induced ALI, and HE staining of lung histology for obese rats showed that renal IRI could notably induce disordered alveolar structure, large numbers of inflammatory cells and red blood cells in the alveolar cavity, and pulmonary interstitial edema, especially in the obesity model rats, and CsA, TIIA, and TIIA+CsA could alleviate lung injury (Figure 2B). In the meantime, the TUNEL results showed that acute renal IRI could induce lung cell apoptosis, and CsA, TIIA, and TIIA+CsA could decrease the quantity of apoptotic cells (*p* < 0.05; Figure 3B), pretreatment with TIIA+CsA was lower than TIIA and CsA. IR (obese) and IR could raise the activity of caspase-3 (*p* < 0.05; Figure 3C), and the cleaved caspase-3 in lung tissues could be raised by IR (obese) and IR (Figure 3D), and using CsA, TIIA, and TIIA+CsA could decrease them, especially in the TIIA+CsA (*p* < 0.05). ALI can induce lung cell apoptosis in a mechanism involving the α2AR/PI3K/Akt pathway in Li et al. (26). Structural damage of lung tissue decreases the external respiratory function; our results showed that the arterial blood pH value, PaO_2_, and PaCO_2_ were decreased in the two groups (IR (obese) and IR), especially in the IR (obese) group (*p* < 0.05; Figure 1). Our results are similar to those by Li et al. (26). The TV, MV, PIF, PEF, and EF50 can be decreased by IR and IR (obese). The Sraw can be raised by IR (obese) and IR (*p* < 0.05), and those index can be improved by giving CsA, TIIA, and TIIA+CsA, especially in the TIIA+CsA (*p* < 0.05; Figure 1).

To date, studies have shown that a variety of anti-inflammatory agents were discovered, including antiapoptotic agents (26), α-MSH (27), and IL-6 inhibitors (28). A previous research found that acute renal IR could induce lung mitochondrial dysfunction (only detected MMP) and that dexmedetomidine could attenuate lung inflammation, apoptosis, and MMP (26). However, using MMP alone to assess the mitochondrial dysfunction is too simple, and more methods and indexes can be used to assess the mitochondrial dysfunction. In our study, we detected MMP, opening of mPTP, mitochondrial ROS, mitochondrial ATP, mtDNA, and mitochondrial dynamics/biogenesis to assess the mitochondrial dysfunction comprehensively. However, no studies have explored the mitochondrial dysfunction in lungs induced by AKI, and no antimitochondrial dysfunction agents have been found to defend against ALI led by AKI in obese rats.

As a universal medicinal herb, modern technology has proved that *Salvia miltiorrhiza* Bge. can eliminate hazardous substances in the blood, promote thrombolysis and the activity of fibrinolytic enzymes, smooth blood vessels, reduce blood viscosity, and protect the cardiovascular system (29).

As the main active ingredient in *Salvia miltiorrhiza* Bge, TIIA can attenuate renal injury induced by renal IR through the downregulation of inflammation and the expression of myeloperoxidase (MPO) and caspase-3 (30). TIIA can decrease the release of cytochrome and the generation of ROS, prevent mPTP opening, reduce the percentage of apoptotic cells, and inactivate caspase-3 (31). Because CsA can inhibit the opening of the mPTP, it has been fundamental in implicating mPTP as a target for protective treatment, which can be improved at the initial stage of reperfusion (31). It is now widely believed that mitochondrial dysfunction (increasing the opening of mPTP and decreasing MMP) plays an important role in raising injury induced by acute renal IRI (18). The opening of mPTP causes the membrane potential to inhibit oxidative phosphorylation and decrease and the swelling of mitochondria. The opening of the mPTP is operated by binding the CyP-D in the mitochondria membrane. Previous researches of the heart showed that CsA defended from IR by binding with CyP protein, independently of anticalcineurin properties, then inhibiting mPTP opening (32). However, to our knowledge, no study has explored whether CsA can be used to inhibit the opening of mPTP to protect renal IRI so as to protect lung mitochondria. Mitochondrial function can be conceptualized as the pathophysiological bridge between kidney-lung interactions during the clinical stage of renal IRI-induced ALI and AKI. Therefore, in this study, we hypothesized that the protective effect of TIIA and CsA on ALI was involved in improving the mitochondrial dysfunction.

As the energy center of cells, mitochondria supply more than 95% ATP (33). Therefore, mitochondria can be seen as a logical target in identifying pathophysiological processes and treatment target in various metabolic diseases. There are many methods for establishing ALI and AKI induced by renal IRI. To explore the mitochondrial relationship between the kidney and the lungs, renal IR rat model was established through removing the right kidney and clamping the left renal artery for 30 min, then reperfusing for 24 h, with pretreatment with TIIA combined with CsA, and then detecting the mitochondrial function. Figure 2C shows that acute renal IRI induced ALI, and using electron microscopy images (×40,000) of rat lung tissue, we could see the abnormal mitochondrial morphology typical of membrane rupture or mitochondrial swellings induced by renal IRI, especially in the obesity model rats, and we could decrease mitochondrial injury through pretreatment CsA, TIIA, and TIIA+CsA, especially with the TIIA+CsA (*p* < 0.05; Figure 2C). IR (obese) and IR could increase the percentage of damaged mitochondria in lung tissues (*p* < 0.05; Figure 2D). In the meantime, IR (obese) and IR could decrease the RCR, especially IR (obese), which could be relieved through pretreatment CsA, TIIA, and TIIA+CsA, especially with the TIIA+CsA (*p* < 0.05; Figure 4). The opening of mPAP (%) and ROS level could be increased by acute renal IR, and using TIIA and CsA could decrease the opening of mPAP (%) and ROS level (*p* < 0.05; Figure 4). MMP (ratio of red/green) and mitochondrial ATP level could be decreased by acute renal IR (*p* < 0.05), and using CsA, TIIA, and TIIA+CsA could decrease the MMP (ratio of red/green) and mitochondrial ATP level, especially with the TIIA+CsA (*p* < 0.05; Figure 4). Our results are similar to liver tissues reported by Luan et al. (34).

The mtDNA copy number in every mitochondria is constant; therefore, we can use the total copy number of mtDNA to evaluate mitochondria quantity in cells (35, 36). Although the mechanism of repairing mtDNA is unknown, mtDNA is adjacent to the respiratory chain, making it more fragile compared with nuclear DNA, because mtDNA is exposed to oxidative stress. In our study, we observed the injury effects of renal IR on mtDNA and CsA, TIIA, and TIIA+CsA had the protective effect. We used the ratio of long and short fragments to evaluate the degree of injury. Our results showed that IR (obese) and IR could decrease the ratio of long/short fragments, especially IR (obese) (*p* < 0.05), and pretreatment with CsA, TIIA, and TIIA+CsA could increase the ratio, especially with the TIIA+CsA (*p* < 0.05; Figure 4). Synthesis of electron transport chain proteins will be inhibited induced by damaged mtDNA, which will inhibit electron transport.

As an important adaptation of exposure to chronic energy deprivation, mitochondrial biogenesis may be modulated through multiple factors, such as Tfam and Nrf1. Nrf1 can facilitate the transcription of many nuclei-encoded mitochondrial proteins, including those involved in respiratory complexes and oxidative phosphorylation. Tfam can increase gene transcription and DNA replication in mitochondria through directly binding to the mitochondrial genome. PGC-1α is a critical transcriptional coactivator and can regulate key factors, including Nrf1 and Tfam and is thought to increase mitochondrial biogenesis (37). When the expression levels of the above genes change, mitochondrial biogenesis will become chaotic. Figure 5 shows that acute renal IR could decrease the PGC-1α, Nrf1, and Tfam expression in the levels of mRNA and protein. TIIA+CsA markedly increased the mRNA and protein expression levels of PGC-1α, Nrf1, and Tfam. Pretreatment with, decreased ROS, a sufficient energy supply (ATP), and increased biogenesis factors may be operated synergistically to result in an improvement in the shortage of the intracellular energy supply (increasing ATP). Normally, harmful stimuli, including energy limitation, oxidative stress, and aging, can induce injury to mitochondria, which are then enclosed by autophagosomes, fused to lysosomes and finally degraded. Autophagy abnormalities in mitochondria can induce the increased levels of damaged mitochondria, resulting in mitochondrial dysfunction (34).

Normally, mitochondria undergo a fusion and fission dynamic process. This dynamic process plays a very important role in maintaining constant changes in the size, network of mitochondria, and shape; they are under the operation of Mfns and Drp1 proteins (38). Our results in Figure 5 showed that the expression levels of Drp1 and Mfns (Mfn1 and Mfn2) changed in opposite directions after acute renal IR, indicating an imbalance of fission-fusion in mitochondria. Mfns and Drp1 were observed to play an important role in mitochondria fusion and mitochondria fission, respectively. We observed a decrease in Drp1 and an increase in Mfn1 and Mfn2 after acute renal IR in the lungs.

The PI3K/Akt/Bad signaling pathway performs an important function in inhibiting mitochondria-mediated apoptosis (20). Based on the close relationship between the PI3K/Akt/Bad pathway and apoptosis, we studied the effect of PI3K in our system. PI3K is a phosphatidylinositol kinase with activities as a serine/threonine-specific protein kinase and a phosphatidylinositol kinase (39). After activation, phosphatidylinositol family members on the cell membrane can be phosphorylated, and the downstream signal molecule Akt can be recruited and activated. Then, activated Akt phosphorylates Ser136/Ser112 residues of the Bad protein (40). Phosphorylated Bad separates from the apoptosis-promoting complex and forms a 14-3-3 protein complex, leading to the inactivation of its apoptosis-promoting function and inhibiting apoptosis (41). Cytochrome c (Cyt-c) release from mitochondria into the cytosol can be induced by mitochondrial dysfunction, and Cyt-c can activate caspase-9 and caspase-3. Activation of either caspase can cleave poly ADP-ribose polymerase (PARP) and induce chromosomal DNA fragmentation (20). Our results showed that TIIA+CsA effectively regulated the expression of apoptotic PI3K/Akt/Bad pathway-related proteins, and CsA, TIIA, and TIIA+CsA could enhance PI3K and p-Akt expression and downregulate the expression of Cyt-c, cleaved caspase-3, and PARP. These results suggested that CsA, TIIA, and TIIA+CsA might improve mitochondrial function and inhibit the lung cells apoptosis induced by acute renal IRI through the PI3K/Akt/Bad signal pathway (Figure 6).

In our study, we separated mitochondria from lung tissues to demonstrate that renal IR promoted a large amount of ROS, which damaged proteins of the electron transport chain and mtDNA, ultimately damaging mitochondrial respiratory function, biogenesis and dynamic function and generating increasing amounts of ROS, especially in obese rats. The swelling of isolated mitochondria was led by the opening of the mPTP after IR. Opening the mPTP may cause a flow back of protons from the mitochondrial membrane space to the matrix, therefore, reducing MMP and ATP synthesis can induce metabolic abnormalities. The reductions in ATP synthesis and MMP were induced by the opening of the mPTP, resulting in the flow back of protons from the mitochondrial membrane space to the matrix, ultimately inducing the metabolic abnormalities and leading to lung cell apoptosis. However, pretreatment with TIIA+CsA can inhibit apoptosis by modulating mitochondrial function through activating the PI3K/Akt/Bad pathway in obese rats.

## Materials and Methods

### Experimental Animals

Experiments were operated on male Sprague-Dawley (SD) rats)Liaoning Changsheng Biotechnology Co., Ltd. [Production License: SCXK(Liao) 2015-0001)], aged 8 weeks and weighing 180–220 g. The rats were housed in cages with controlled conditions of 45–65% humidity and 20 ± 3°C and with 12-h cycle of light/dark (lights on 06:00 h) and were fed with a pellet diet and water *ad libitum*.

### Ethical Statement

Animal operating procedures and the experimental design were approved by the Ethical Committee of Animal Handling (2019019) of Liaoning University of Traditional Chinese Medicine, Shenyang, China and abided by the guidelines of the Care and Use of Laboratory Animals published by the US National Institutes of Health, and we made every effort to decrease the number of rats utilized and their suffering. In the meantime, we did our utmost to supply the better surroundings to the rats during research.

### Drugs

TIIA (Injection of Sulfotanshinone Sodium, 10 mg each) was supplied by No. 1 Biochemical Pharmaceutical Co., Ltd in Shanghai. We used the deionized water to dissolve TIIA in order to obtain the 5-mg/ml stock solution. CsA (20 mg each) was obtained from Solarbio Science & Technology Co., Ltd, in Beijing. We used dimethylsulfoxide (DMSO) (0.1%) to dissolve CsA to obtain 2.5 mg/ml stock solution, which would be further diluted to obtain the appropriate concentration.

### Animal Groupings and Methods of Drug Dosing

We randomly divided 120 rats into six groups, including the Sham operation, the IR, the IR (obese), the TIIA, the CsA, and the TIIA+ CsA group, with 20 rats in each group. All rats in the six groups were fed with general maintenance food for 2 weeks for the purpose of adapting to the environment. Then, the Sham group and the IR group were still fed with maintenance food for 8 weeks, but the other four groups were fed with HFD for 8 weeks. Sham group, the IR, and IR (obese) groups rats were given intraperitoneal injection of deionized water for 2 weeks. We gave 10 mg/(kg/day) TIIA (intraperitoneal injection) for 2 weeks before renal IR in the rats of TIIA group. We gave the 5-mg/kg CsA (intraperitoneal injection) 30 min before renal IR in the rats of CsA group, and the 10 mg/(kg/day) TIIA (intraperitoneal injection) for 2 weeks before renal IR+ 5 mg/kg CsA (intraperitoneal injection) 30 min before renal IR in the rats of TIIA+ CsA group. The components of the HFD included 25% total fat containing 18% protein, 11% unsaturated fat, 13% fiber, 44% carbohydrate, ash, and other ingredients (42). We chose the rats that increased 30% body weight for further research (43).

### Surgical Procedure

We used the thiopental sodium (120 mg/kg) to anesthetize rats through intraperitoneal injection, and we pinched the rats paw and tail to evaluate the anesthetic effect. We opened the abdomen to expose the right kidney, and then dissected the renal pedicle to expose the renal vessels. 3–0 silk suture was used to ligate the blood vessel, and then we removed the right kidney. We exposed the left kidney, and used an arterial clamp to clamp off left renal artery for 30 min to build ischemia. With the 30-min ischemia, we removed the arterial clamp, and the tissue was reperfused for 24 h. The left kidneys were observed for 15 min to ensure normal blood reperfusion, which was shown by the color change of kidney to red again (44). The wound was closed with 3–0 silk suture. The rats were placed on a heating pad to maintain the 37°C body temperature throughout the whole experimental procedure. Sham-operated rats received the same surgical procedures without clamping off renal artery for 30 min (23).

### Arterial Blood Gas Analysis

Arterial blood samples were used for arterial blood gas analysis. Arterial blood (0.5 ml) was obtained from the abdominal aorta, and the pH, partial pressure of oxygen (PaO_2_), and partial pressure of carbon dioxide (PaCO_2_) were measured with a blood gas analyzer (Beckman Coulter, Inc., USA).

### Pulmonary Function Analysis

After the device [DSI's FinePointe Software and FinePointe Non-invasive Airway Mechanics (NAM) DSI, Inc., USA] was connected, rats were introduced into the double-lumen chamber and fixed to minimize the stress response. Data were collected after the double-lumen tube was stabilized. The data were collected and measured for 5 min each time. The rats were kept quiet before being measured. We chose tidal volume (TV), minute ventilation (MV), peak inspiratory force (PIF), peak expiratory force (PEF), exhale force Metaphase (EF50), and specific resistance of the airway (SRaw).

### Histological Assessment of the Lung Using HE Staining

HE staining was performed as previously reported. After paraffin embedding, lung tissues were sectioned into 5-μm-thick sections (45). After immersion in paraformaldehyde (4%) for 24 h and then dehydration with ethanol (70%), we used H&E staining to stain the lung tissues and then visualized lung tissues with light microscopy. As previously reported, a scoring system was used to evaluate the histopathological injury (46). The injury degree of lung tissues was scored as follows (26): 0 grade, normal pulmonary tissues; 1 grade, mild or moderate infiltrations of leukocyte and neutrophil and interstitial congestion; 2 grade, formatting perivascular edema, partial leukocyte infiltration, and moderate infiltrations of neutrophils and leukocytes; and 3 grade, severe destruction in lung tissues and massive infiltration of neutrophils and leukocytes.

### Apoptosis Assessment of the Lung Using TUNEL

We used the terminal deoxynucleotidyl transferase-mediated dUTP nick end-labeling (TUNEL) assay [*In Situ* Cell Death Detection Kit (Roche, Germany)] to detect apoptosis. As described in the HE staining section (45), we used 5-μm-thick sections to perform TUNEL staining. After deparaffinization and rehydration, protease K (10 μg/ml) was added to the sections for 15 min. The samples were supplemented with fresh TUNEL reaction mixture and were incubated at 37°C for 60 min with the dark. After washing, we used 0.1 μg/ml 4′,6-diamidino-2-phenylindole (DAPI) (Beyotime, China) to stain the cell nuclei. We used the fluorescence microscope (Canon, Japan) to analyze the samples in a drop of phosphate-buffered saline (PBS). We used a blinded manner to observe eight random visual fields per animal with ×200 magnification to calculate and analyze the number of TUNEL-positive cells per high-power field.

### Caspase-3 Activity

We used the fluorescent caspase-specific substrate AcDEVD-7-pNA (Solarbio) to detect the caspase-3 activity. The lung tissue proteins (10 mg) were added to reaction buffer for incubation (37°C) for 2 h. We used the fluorimeter (405 nm) to quantify the enzyme-catalyzed release.

### Using Electron Microscope to Observe Mitochondria

Lung tissues were obtained immediately after anesthesia and cut into small pieces (1 mm^3^). After fixing the specimens with 2% glutaraldehyde at 4°C, we used phosphate buffer (0.1 mol/L) to wash the samples, which was then fixed with 1% osmium tetroxide and then stained with 1% aqueous uranyl acetate. We used capsules including embedding medium to place the specimens 70°C for approximately 48 h. Uranyl acetate and alkaline lead citrate were used to stain specimen sections then to observe under electron microscope (HITACHI H-7650, Tokyo, Japan).

### Preparation of Lung Mitochondria Suspension

As previously studied in the liver (34), rats were anesthetized, and then the lung was harvested and placed in a pH 7.4 ice-cold isolated buffer (10 mM Tris-HCl, 250 mM sucrose, and 1 mM EDTA). After trimming, lung tissues were rinsed with a homogenizer in an isolation buffer, and 50–100 mg of tissue was weighed. For preserving mitochondria integrity, the whole isolation process was performed at 4°C. Following centrifugation at 700× *g* for 10 min, the supernatant was collected and the samples were centrifuged at 7,000× *g* for 10 min again. Then, we discarded the supernatant and washed the mitochondria pellet with 5 ml of isolation buffer, centrifuging twice in 7,000× *g* for 10 min. We obtained a clean mitochondria solution and preserved it in mitochondrial preservation solution (10 mM KH_2_PO_4_, 5 mM HEPES, 2 mM MgCl_2_, 1 mM EDTA, 100 mM KCl, and 20 mM sucrose) to get a mitochondria suspension (5 mg/ml protein), which was placed on ice for immediate use. We measured the protein concentration of mitochondrial suspensions using the bicinchoninic acid (BCA) reagent box (Beyotime, Shanghai, China) and to ensure the protein concentrations were between 100 and 1,000 μg/ml. Mitochondria suspension was used to detect the mitochondrial membrane potential (MMP), the opening of the mPTP, reactive oxygen species (ROS), respiratory control rate (RCR), and adenosine triphosphate (ATP) synthesis.

### Measurement of MMP

We detected the MMP using the mitochondrial membrane potential assay kit (Beyotime, Shanghai, China). (1) Diluting five times the JC-1 dyeing buffer into 1×; (2) then, we added five-diluted JC-1 working solution (0.9 ml) into purified mitochondria (0.1 ml) with the protein level 100–1,000 μg/ml; and (3) MMP was detected by fluoroenzyme-labeled reagent. We added mixed solution (1 ml) to fluoroenzyme-labeled assay, with the 530-nm emission wavelength and 490-nm excitation wavelength, and then the ratio of the red/green was calculated.

### Measurement of ATP

We used the luciferase-based luminescence enhanced ATP assay kit (Beyotime, Shanghai, China) to measure the level of adenosine triphosphate (ATP) in the isolated mitochondria. We incubated isolated mitochondria (1 mg/ml) into the 0.5-mL respiration buffer (2.5 mM succinate, 2.5 mM malic acid, and 2.5 mM ADP) for 10 min. We used the SpectraMax Paradigm Multi-Mode Microplate Reader (Molecular Devices, Sacramento, CA, USA) to detect the ATP concentrations.

### The Opening of mPTP

The opening of the mPTP was measured by detecting the A540 absorbance of mitochondria exposed to 250 μM CaCl_2_ (“+”: treatment with Ca^+^; “–”: treatment without Ca^+^). Purified Mitochondrial Membrane Pore Channel Colorimetric Assay kit (GENMED, Shanghai, China) was used for detection; 200 μmol/L CaCl_2_ was used to induce mPTP opening. An ultramicro microporous plate spectrophotometer (Biotek, USA) was used to read the value of optical density (OD) from 0 to 10 min at 520 nm. The OD decrease reflected the mPTP opening. The OD value noted at the onset of the experiment (0 min) represented the minimum optical density (min OD); the OD value noted at the end of the experiment (10 min) represented the maximum optical density (min OD). The min/max OD was negatively associated with the extent of MPTP opening (34, 47).

### Measurement of ROS

ROS was detected using a Multi-Mode Microplate Reader with a fluorescent probe of 2′,7′-dichlorodihydrofluorescein diacetate (DCFH-DA).

### Measurement of RCR

We used the RCR (ratio of state III and state IV) to assess the integrity of isolated mitochondria, oxidative phosphorylation, and respiratory chain function. RCR was assessed using an Oxytherm Clark-type oxygen electrode (OXYT1/ED; Hansatech Instruments, Norfolk, UK). Sixty micrograms mitochondria (separated from the lung) were put into the oxytherm chamber containing pH 7.2 respiration buffer (0.1% BSA, 125 mM KCl, 20 mM HEPES, 2 mM MgCl_2_, and 2.5 mM KH_2_PO_4_) and stirring respiration buffer at 37°C to ensure that all of the respiration buffer contained equal amounts of mitochondria. For each state of respiration, the slope of the response of mitochondria to consecutive administrations of respiration substrates was defined as rate of oxygen consumption, as previously reported (48).

### Measurement of Damaged mtDNA

The injured mtDNA was calculated through the ratio of long and short fragments using real-time quantitative polymerase chain reaction (RT-qPCR). (1) DNA isolation: total lung DNA was extracted by the Genomic-tip 20/G kit (Qiagen, Valencia, CA, USA). The quantification of the PCR products or purified DNA was performed fluorometrically using the Picogreen dsDNA reagent (Invitrogen, Milan, Italy). (2) RT-qPCR was performed on lung DNA extracts as previously reported (35) using the following modification: the PCR amplification was performed using the Ranger DNA Polymerase with appropriate premixes (Bioline Ltd., London, UK). The two pairs (mtDNA long fragment and short fragment) of primers (General Biosystems, Anhui, China) are shown in Table 2. (3) For amplification of the mtDNA long fragment, the standard thermocycler program included an initial denaturation at 94°C (1 min), 94°C (15 s) for 18 cycles, 65°C (12 min), and a final extension at 72°C (10 min). The short fragment of the mtDNA was amplified by the same condition, except that the extension temperature was regulated to 60°C.

**Table 2:** Sequence of primers for RT-PCR and long PCR.

**Target gene**	**Primer sequence**	**Size (bp)**	**Tm (°C)**
Mfn1	Forward: 5′-GGGAAGACCAAATCGACAGA-3′	152	57
	Reverse: 5′-CAAAACAGACAGGCGACAAA-3′		57
Mfn2	Forward: 5′-GAGAGGCGATTTGAGGAGTG-3′	165	58
	Reverse: 5′-CTCTTCCCGCATTTCAAGAC-3′		56
Drp1	Forward: 5′-GCCCGTGGATGATAAAAGTG-3′	215	56
	Reverse: 5′-TGGCGGTCAAGATGTCAATA-3′		56
PGC-1α	Forward: 5′-GGACGAATACCGCAGAGAGT-3′	201	59
	Reverse: 5′-CCATCATCCCGCAGATTTAC-3′		56
Nrf1	Forward: 5′-AAACCGAACACATGGCTACC-3′	168	58
	Reverse: 5′-CTGCCGTGGAGTTGAGTATG-3′		58
Tfam	Forward: 5′-TCACCTCAAGGGAAATTGAAG-3′	241	55
	Reverse: 5′-CCCAATCCCAATGACAACTC-3′		56
Long fragment	Forward:5′-AAAATCCCCGCAAACAATGACCACCC-3′	13,400	72
	Reverse: 5′-GGCAATTAAGAGTGGGATGGAGCCAA-3′		72
Shrot fragment	Forward: 5′-CCTCCCATTCATTATCGCCGCCCTGC-3′	235	60
	Reverse: 5′-GTCTGGGTCTCCTAGTAGGTCTGGGAA-3′		60
GAPDH	Forward: 5′- AGGTCGGTGTGAACGGATTTG−3′	20	58
	Reverse: 5′- GGGGTCGTTGATGGCAACA-3′		58

### RNA Extraction and cDNA Synthesis

We isolated total genome RNA with TRIzol reagent (Invitrogen, Carlsbad, CA, USA). Spectrophotometry (260 nm) was used to assess the quality of isolated RNA. Reverse transcription was performed with 1 μg total RNA and an M-MLV Reverse Transcriptase Kit (Promega A3500; Promega, Madison, WI, USA). Briefly, the 40-μl total reaction volume was used in a Veriti 96 Well Thermal Cycler Long PCR system (Applied Biosystems, Foster City, CA, USA) according to the following reaction procedure: 72°C (3 min), 42°C (90 min), 70°C (15 min) and held at 4°C.

### Real-Time qPCR

We used the RT-qPCR to detect the copy numbers of the transcription levels of specific genes with cDNA templates. PCR was performed on a Rotor-Gene Q Sequence Detection System (QIAGEN, Germany) using SYBR Premix Ex TaqII (TakaraBioINC) (49). The PCR was conducted in a 20-μl system (1 μl synthetic cDNA + 10 μl SYBR Premix Ex Taq II + 0.5 μM primers), with the following procedure: 95°C (10 min); 95°C (10 s), 40 cycles, 60 °C (15 s); 72 °C (20 s); and 72 °C (10 min). The values were calculated with GAPDH as the internal control (50). Two pairs of PCR primer sequences used in our study are shown in Table 2.

### Protein Detection

Western blot was used to detect target protein. Total proteins were extracted from tissues using RIPA Lysis Buffer. Protein concentration was measured using a BCA Protein Assay Kit. To examine the expression of proteins, the same amount of total protein was loaded on an 8–12% sodium dodecyl sulfate-polyacrylamide gel electrophoresis (SDS-PAGE) gel. Separated proteins were then transferred onto PVDF membranes. After being blocked in skim milk solution, the membrane was incubated overnight separately with the antibodies anti-GAPDH, anti-PI3K, anti-p-Akt, anti-Akt, anti-p-Bad, anti-Bad, anti-Bax, anti-Bcl-2, anti-Cyt-c, anticaspase-3, anticleaved-caspase-3, anti-PARP, anti-Drp1, anti-Mfn1, anti-Mfn2, anti-PGC-1, anti-NRF1, and anti-TFam (antibodies are shown in Table 3). Subsequently, the membrane was incubated with secondary HRP-conjugated goat antirabbit antibodies (Santa Cruz Biotechnology). Proteins were visualized using an enhanced chemiluminescence kit from Thermo Fisher Scientific (Massachusetts, USA). ImageJ software (Alpha View SA) was used to perform densitometric analysis.

**Table 3:** Antibodies used in the study.

**Antibodies**	**Manufacturer**	**Catalog no**.	**Observed MW**	**Dilution**
Anti-PI3K	Proteintech	67071-1-1g	110 KDa	1:10,000
Anti-p-Akt	Proteintech	66444-1-1g	62 KDa	1:10,000
Anti-Akt	Proteintech	10176-2-AP	56 KDa	1:5,000
Anti-p-Bad	Cell signaling technology	5284S	23 KDa	1:1,000
Anti-Bad	Proteintech	10435-1-AP	18 KDa	1:2,500
Anti-Bcl-2	Proteintech	26593-1-AP	26 KDa	1:2,500
Anti-Bax	Proteintech	50599-2-1g	26 KDa	1:10,000
Anti-Caspase-3	Proteintech	19677-1-AP	32 KDa	1:2,000
Anti-cleaved-Caspase-3	Abcam	ab49822	17 KDa	1:500
Anti-PARP1	Proteintech	13371-1-AP	89 KDa	1:2,000
Anti-Cyt-c	Proteintech	12245-1-AP	13 KDa	1:2,000
Anti-Mfn1	Proteintech	13798-1-AP	86 KDa	1:1,000
Anti-Mfn2	Proteintech	12186-1-AP	86 KDa	1:5,000
Anti-Drp1	Proteintech	10656-1-AP	27 KDa	1:4,000
Anti-PGC1a	Proteintech	66369-1-1 g	100 KDa	1:5,000
Anti-Nrf1	Proteintech	12482-1-AP	67 KDa	1:2,500
Anti-Tfam	Proteintech	22586-1-AP	25 KDa	1:5,000
Anti-GAPDH	Proteintech	60004-1-1g	36 KDa	1:10,000

### Statistical Analysis

Statistical analysis was operated by the SPSS statistical package (Version 17.0, SPSS Inc. Chicago, IL, USA). The mean ± standard deviation was used to express the data. The one-way analysis of variance (ANOVA) was used to compare among six independent groups. The two-to-two comparison among groups was used to analyze the variance, and Tukey's *t* test was used for multiple comparisons between six groups. We regarded *p* < 0.05 as having statistically significant difference.

## Conclusions

Our results demonstrated that lung mitochondrial dysfunction was induced in the process of renal IR, especially in the obese rats. Mitochondrial dysfunction can be seen as the direct pathophysiological kidney-lung interactions during the stage of AKI and ALI induced by renal IR. During this process, lung mitochondrial function was impaired, dynamics was altered, and biogenesis was inhibited. ROS overproduction led to mtDNA damage and a significant decrease of MMP followed by a reduction of intracellular ATP. Thus, TIIA+CsA can be seen as a protective agent, which can attenuate lung apoptosis *via* modulating mitochondrial function by activating PI3K/Akt/Bad pathway in obese rats. But in our study, considering of the combination therapy, we did not use the PI3K/Akt/Bad pathway inhibitor, so the mechanism of drug intervention cannot be fully revealed. In the future, we will explore the other protective mechanism of TIIA+CsA and the signaling pathway of apoptosis. These results may be a promising protective strategy for managing obesity-related AKI and ALI. However, this application needs further large-scale experimental and clinical studies.

## Data Availability Statement

The original contributions presented in the study are included in the article/supplementary material, further inquiries can be directed to the corresponding author/s.

## Ethics Statement

The animal study was reviewed and approved by Animal operating procedures and the experimental design were approved by the Ethical Committee of Animal Handling (2019019) of Liaoning University of Traditional Chinese Medicine.

## Author's Note

We assess as a part of our study about heart apoptosis have been uploaded on a pre-print server which can be accessed at https://www.researchsquare.com/article/rs-77027/v1, and it has been submitted to another journal. Because our group have studied about the adjacent organs (the heart and lung) apoptosis induced by renal IR, and the materials and methods have many similarities which is hard to avoid, but the detection index are different from each other. In this manuscript we researched the lung apoptosis.

## Author Contributions

HT and X-lJ wrote the manuscript and researched data. YL, H-hX, and NS selected the rats and extracted blood. M-jC and X-mY dealed with the figures. Y-rC detected related index. G-lY and L-qJ contributed to the discussion and reviewed the manuscript. Rats model is builded by H-hX and M-jC. Editorial support (in the form of writing assistance, including development of the initial draft based on author input, assembling tables and figures, collating authors comments, grammatical editing, and referencing) was provided by HT, Z-mL, H-hX, and YL. The translator of English was provided by HT, X-lJ, and J-sK.

## Conflict of Interest

The authors declare that the research was conducted in the absence of any commercial or financial relationships that could be construed as a potential conflict of interest.

## Abbreviations

IR: ischemia-reperfusion
AKI: acute kidney injury
ALI: acute lung injury
CsA: cyclosporine A
TIIA: tanshinone IIA
HE: hematoxylin-eosin
TUNEL: terminal deoxynucleotidyl transferase-mediated dUTP nick end-labeling
MMP: mitochondrial membrane potential
ATP: adenosine triphosphate
ROS: reactive oxygen species
RCR: respiration controlling rate
mPTP: mitochondrial permeability transition pore
OD: optical density
CyP-D: cyclophilin D
DMSO: dimethylsulfoxide
HFD: high-fat diet
NF-κB: nuclear translocation of the transcription factors
SD: Sprague-Dawley
PBS: phosphate-buffered saline
DAPI: 4′, 6-diamidino-2-phenylindole
DCFH-DA, 2′: 7′-dichlorofluorescein diacetate
RT-qPCR: real-time quantitative PCR
HE: hematoxylin-eosin
PaO_2_: partial pressure of oxygen
PaCO_2_: partial pressure of carbon dioxide
NAM: Non-invasive Airway Mechanics
TV: tidal volume
MV: minute ventilation
PIF: peak inspiratory force
PEF: peak expiratory force
EF50: exhale force metaphase
Sraw: specific resistance of airway
BCA: bicinchoninic acid
qPCR: quantitative polymerase chain reaction
mPTP: mitochondrial permeability transition pore
mtDNA: mitochondrial DNA
MPO: myeloperoxidase
IRI: ischemia-reperfusion injury
ANOVA: analysis of variance
PI3K: phosphoinositide-3 kinase
Cyt-c: cytochrome c
PARP: poly ADP-ribose polymerase.
